# Cancer incidence in persons with type 1 diabetes: a five-country study of 9,000 cancers in type 1 diabetic individuals

**DOI:** 10.1007/s00125-016-3884-9

**Published:** 2016-02-29

**Authors:** Bendix Carstensen, Stephanie H Read, Søren Friis, Reijo Sund, Ilmo Keskimäki, Ann-Marie Svensson, Rickard Ljung, Sarah H Wild, Joannes J Kerssens, Jessica L Harding, Dianna J Magliano, Soffia Gudbjörnsdottir, on behalf of the Diabetes and Cancer Research Consortium

**Affiliations:** Steno Diabetes Centre, Gentofte, Denmark; Usher Institute of Population Health Sciences & Informatics, University of Edinburgh, Teviot Place, Edinburgh, EH8 9AG Scotland UK; Institute of Cancer Epidemiology, Danish Cancer Society, Copenhagen, Denmark; Centre for Research Methods, Department of Social Research, University of Helsinki, Helsinki, Finland; Division of Health and Social Services, National Institute for Health and Welfare, Helsinki, Finland; Department of Medicine, Sahlgrenska University Hospital, University of Gothenburg, Gothenburg, Sweden; Institute of Environmental Medicine, Karolinska Institutet, Stockholm, Sweden; Information Services, NHS National Services Scotland, Edinburgh, Scotland UK; Department of Clinical Diabetes and Epidemiology, Baker IDI Heart and Diabetes Institute, Melbourne, Australia

**Keywords:** Cancer incidence, Cancer rate ratio, Cancer subtypes, Diabetes duration

## Abstract

**Aims/hypothesis:**

An excess cancer incidence of 20–25% has been identified among persons with diabetes, most of whom have type 2 diabetes. We aimed to describe the association between type 1 diabetes and cancer incidence.

**Methods:**

Persons with type 1 diabetes were identified from five nationwide diabetes registers: Australia (2000–2008), Denmark (1995–2014), Finland (1972–2012), Scotland (1995–2012) and Sweden (1987–2012). Linkage to national cancer registries provided the numbers of incident cancers in people with type 1 diabetes and in the general population. We used Poisson models with adjustment for age and date of follow up to estimate hazard ratios for total and site-specific cancers.

**Results:**

A total of 9,149 cancers occurred among persons with type 1 diabetes in 3.9 million person-years. The median age at cancer diagnosis was 51.1 years (interquartile range 43.5–59.5). The hazard ratios (HRs) (95% CIs) associated with type 1 diabetes for all cancers combined were 1.01 (0.98, 1.04) among men and 1.07 (1.04, 1.10) among women. HRs were increased for cancer of the stomach (men, HR 1.23 [1.04, 1.46]; women, HR 1.78 [1.49, 2.13]), liver (men, HR 2.00 [1.67, 2.40]; women, HR 1.55 [1.14, 2.10]), pancreas (men, HR 1.53 [1.30, 1.79]; women, HR 1.25 [1.02,1.53]), endometrium (HR 1.42 [1.27, 1.58]) and kidney (men, HR 1.30 [1.12, 1.49]; women, HR 1.47 [1.23, 1.77]). Reduced HRs were found for cancer of the prostate (HR 0.56 [0.51, 0.61]) and breast (HR 0.90 [0.85, 0.94]). HRs declined with increasing diabetes duration.

**Conclusion:**

Type 1 diabetes was associated with differences in the risk of several common cancers; the strength of these associations varied with the duration of diabetes.

**Electronic supplementary material:**

The online version of this article (doi:10.1007/s00125-016-3884-9) contains peer-reviewed but unedited supplementary material, which is available to authorised users.

## Introduction

Persons with diabetes have an approximately 20–25% higher cancer incidence compared with persons without diabetes, though this varies by cancer site [1]. In particular, persons with diabetes have been shown to have an elevated incidence of liver, pancreatic, colorectal, endometrial and kidney cancer [1]. Conversely, a decreased risk of prostate cancer has been reported in men with diabetes [2–5].

The association between type 1 diabetes and cancer is not well described. The majority of previous studies assessing the link between diabetes and cancer have not made a distinction between the two major types of diabetes. As type 2 diabetes is far more prevalent than type 1 diabetes, study populations have been primarily composed of persons with type 2 diabetes, thus hindering the generalisation of findings to persons with type 1 diabetes. It is possible that the relationship between type 1 diabetes and cancer is different from that observed between type 2 diabetes and cancer as a result of differences in the underlying disease characteristics, drug therapies and patterns of risk factors, such as obesity.

The observed excess risk of cancer in persons with diabetes may, to some extent, be a consequence of anti-diabetic drug therapies, such as exogenous insulin [6]. There is some evidence that insulin-treated patients have a higher cancer incidence compared with non-insulin-treated patients [7]. If exogenous insulin use is associated with increased cancer risk, then an excess risk of cancer should be apparent in persons with type 1 diabetes, and this excess risk could potentially be larger than that observed in persons with type 2 diabetes. Alternatively, hyperglycaemia has also been suggested as a possible mechanism through which diabetes is associated with cancer [8, 9]. If hyperglycaemia does contribute to the observed elevated risks of cancer among persons with type 2 diabetes then similar associations would be expected for both type 1 diabetes and type 2 diabetes patients.

The small number of existing studies investigating cancer occurrence among persons with type 1 diabetes [10–13] have been underpowered to provide accurate risk estimates for site-specific cancers and have subsequently reported heterogeneous findings [14]. Further limitations of previous studies include unrepresentative samples, difficulties in defining type 1 diabetes, short follow up and inadequate evaluation of the potential influence of ascertainment bias on diabetes duration.

In this large multinational study, we compared cancer incidence among persons with type 1 diabetes and the general population using population-based registries in five countries. The effect of diabetes duration on the association with cancer was also explored.

## Methods

### Data sources

The data sources for this project were compiled from the following five countries: Australia, Denmark, Finland, Scotland and Sweden. Details of each country’s source of diabetes, cancer and mortality data, as well as details of ethical approval, are provided in the electronic supplementary material (ESM). The study periods for each country were: Australia, 2000–2008; Denmark, 1995–2012; Finland, 1972–2010; Scotland, 1995–2011; and Sweden, 1987–2012. ICD-10 (www.who.int/classifications/icd/en/) and ICD-7 codes were used to classify individual cancer diagnoses.

In each country, persons with type 1 diabetes were defined as persons who were recorded with a diagnosis of diabetes below the age of 40 years. Follow up time and the number of cancers in persons with type 1 diabetes were classified by country, sex, age and calendar time (in 1 year categories) and by diabetes duration (subdivided at 0, 1, 2, 5, 10, 15 and 30 years).

Population size by sex, age and calendar time were obtained from the respective national statistics and used to estimate person-years at risk for each country.

### Statistical methods

#### Definition of outcome

We followed persons with type 1 diabetes from the study start date or the date of diabetes diagnosis, whichever was latest, and excluded patients with a pre-existing cancer diagnosis. All individuals were followed until the first cancer occurrence, death or study end date, whichever came first.

The incidence of cancer was defined by the first primary cancer only and the date of cancer diagnosis was retrieved from the respective cancer registries.

As the prevalence of type 1 diabetes is low (<1%), we chose to compare cancer incidence in persons with type 1 diabetes with that in the total background population rather than in a non-diabetic population. For convenience, we also chose to measure follow up among persons with type 1 diabetes up to the date of death or the end of study, and ignored the date of cancer diagnosis to avoid the recalculation of person-years at risk for each cancer type. This approach overestimated the follow up time for persons with type 1 diabetes by <5% for all cancers combined and by much less than 5% for cancer at specific sites (see http://bendixcarstensen.com/DMCa/T1D/T1D-Ca.pdf (accessed 8 January 2016), section 8.4.2, for sensitivity analyses). We censored follow up at cancer diagnosis in the analysis of all cancers and in the grouping of non-sex-specific cancers.

#### Statistical models

For each cancer site and sex, data were classified by country, age and period of follow up, date of birth (i.e. cohort), and general population vs diabetes patients (for disease duration). We fitted an age–period–cohort model to the cancer incidence data separately for each sex and for each studied cancer site. The models were fitted using cubic spline terms for age, period (date of follow up) and cohort (date of birth), and using the events as outcome and the natural log of person-years as the offset in a Poisson regression model [15, 16]. Spline knots were chosen so the number of cases between consecutive knots was the same across each of the following variables: age, period and cohort.

We assumed a common effect of type 1 diabetes in the first model and a set of common duration terms in the second model. Thus, the model for the cancer incidence rate (λ_*napcd*_) was:

$$ \log \left({\lambda}_{napcd}\right) = {f}_n(a) + {g}_n(p) + {h}_n(c) + {\delta}_d, $$

where *n* denotes country, *a* denotes age, *p* denotes period (calendar time), *c* denotes cohort (date of birth) and *d* denotes diabetes duration as evaluated at the start of each small interval. In the simple model, *d* only took the values ‘general population’ or ‘type 1 diabetic patients’. In the extended analysis, it took the values ‘general population’ or 0, 1, 2, 5, 10, 15 or 30 years, although the latter values were present in a slightly smaller dataset because the duration of type 1 diabetes was not available for prevalent cases at the study start times for Denmark and Australia. The variable ‘duration of type 1 diabetes’ was only incorporated into site-specific models in which there were at least 200 cancer cases among persons with type 1 diabetes for each sex, with the addition of kidney cancer cases because of its established association with type 2 diabetes.

The model was thus a proportional hazards model which assumed that the HR of cancer among persons with type 1 diabetes relative to the general population was constant across age and calendar time categories. This is the same assumption that underlies a traditional standardised mortality ratio analysis, but we modelled the population cancer incidence rates using smooth terms instead of using empirical rates in small intervals. This was done because a fairly large fraction of the follow up occurred in age groups in which population rates were unstable, particularly for rare cancer types. Our approach stabilised population rates by imposing the reasonable assumption that rates of cancer varied smoothly over time in both the total population and persons with type 1 diabetes.

We tested whether the specific age cut-off of 40 years was appropriate by including an interaction between age at diagnosis (<30, 30–35, 35–40) and the HR of cancer between those with type 1 diabetes and the general population. We also fitted an extended model with separate type 1 diabetes HR for each country and tested this against the model with a common HR across countries to assess heterogeneity.

All calculations and plots were carried out in R, version 3.1.2 [17], and using the R Epi package [18]. A complete account of all data manipulations and analyses is available at http://bendixcarstensen.com/DMCa/T1D/T1D-Ca.pdf (accessed 8 January 2016).

## Results

Among persons with type 1 diabetes, there were a total of 9,149 first incident cancers in 3.9 million person-years of follow up. The distribution of cancer by country, sex and site are presented in Table 1 (also included as ESM Table 1 for completeness). Histograms of events and person-years by age, calendar time and duration for each country (and the total) are presented in ESM Fig. 1.

**Table 1:** Person-years and number of cancer cases in persons with diabetes diagnosed at age under 40 years by sex, country and cancer

Cancer site	Men						Women						Total
	AU	DK	FI	SC	SE	Subtotal	AU	DK	FI	SC	SE	Subtotal	
Person-years (×1,000)^a^	255.5	255.6	547.9	178.7	737.5	1,975.1	255.4	289.0	636.8	145.5	631.8	1,957.7	4,064.0
All sites	504	401	1,000	253	1,882	4,040	600	641	1,408	280	2,180	5,109	9,149
Non-sex-specific	443	352	832	222	1,480	3,329	364	351	680	146	1,122	2,663	5,992
Person-years (×1,000)^b^	255.5	258.9	553.8	179.5	746.0	1,993.7	255.4	293.9	648.0	146.8	645.2	1,989.3	3,983.0
Oesophagus	9	4	16	9	29	67	3	1	5	7	14	30	97
Stomach	12	6	47	5	64	134	13	14	50	4	39	120	254
Colon	NA	26	56	18	173	273	NA	16	66	9	119	210	483
Rectum	NA	13	46	15	84	158	NA	10	41	6	57	114	272
Colorectal	61	39	102	33	257	492	60	26	107	15	176	384	876
Liver	14	9	34	12	44	113	7	6	8	3	17	41	154
Pancreas	19	15	54	7	52	147	8	9	41	5	30	93	240
Lung	41	39	119	37	134	370	29	38	58	19	128	272	642
Melanoma	86	21	60	16	122	305	72	59	59	15	103	308	613
Breast	–	–	–	–	–	–	184	184	546	99	710	1,723	1,723
Cervix	–	–	–	–	–	–	11	48	36	14	85	194	194
Endometrium	–	–	–	–	–	–	25	40	97	12	149	323	323
Ovary	–	–	–	–	–	–	22	25	80	11	114	252	252
Prostate	41	12	148	11	341	553	–	–	–	–	–	–	553
Testis	22	37	23	20	57	159	–	–	–	–	–	–	159
Kidney	21	23	67	14	62	187	15	15	47	4	37	118	305
Bladder	12	17	49	11	124	213	2	7	16	6	35	66	279
Brain/central nervous system	17	31	35	18	79	180	13	42	32	28	98	213	393
Thyroid	15	6	18	4	15	58	45	29	104	12	51	241	299
Non-Hodgkin’s lymphoma	30	26	56	21	111	244	19	14	46	8	56	143	387
Hodgkin’s lymphoma	11	11	14	NA	20	56	2	4	9	NA	9	24	80
Multiple myeloma	2	4	14	3	26	53	2	5	8	0	13	28	81
Leukaemia	NA	14	39	13	38	104	NA	16	33	9	28	86	190

^a^Follow up only to the first primary tumour of any kind or end of study

^b^Follow up until death or end of study; used for analysis of specific sites

AU, Australia; DK, Denmark; FI, Finland, SC, Scotland; SE, Sweden; NA, data not available

**Fig. 1:**
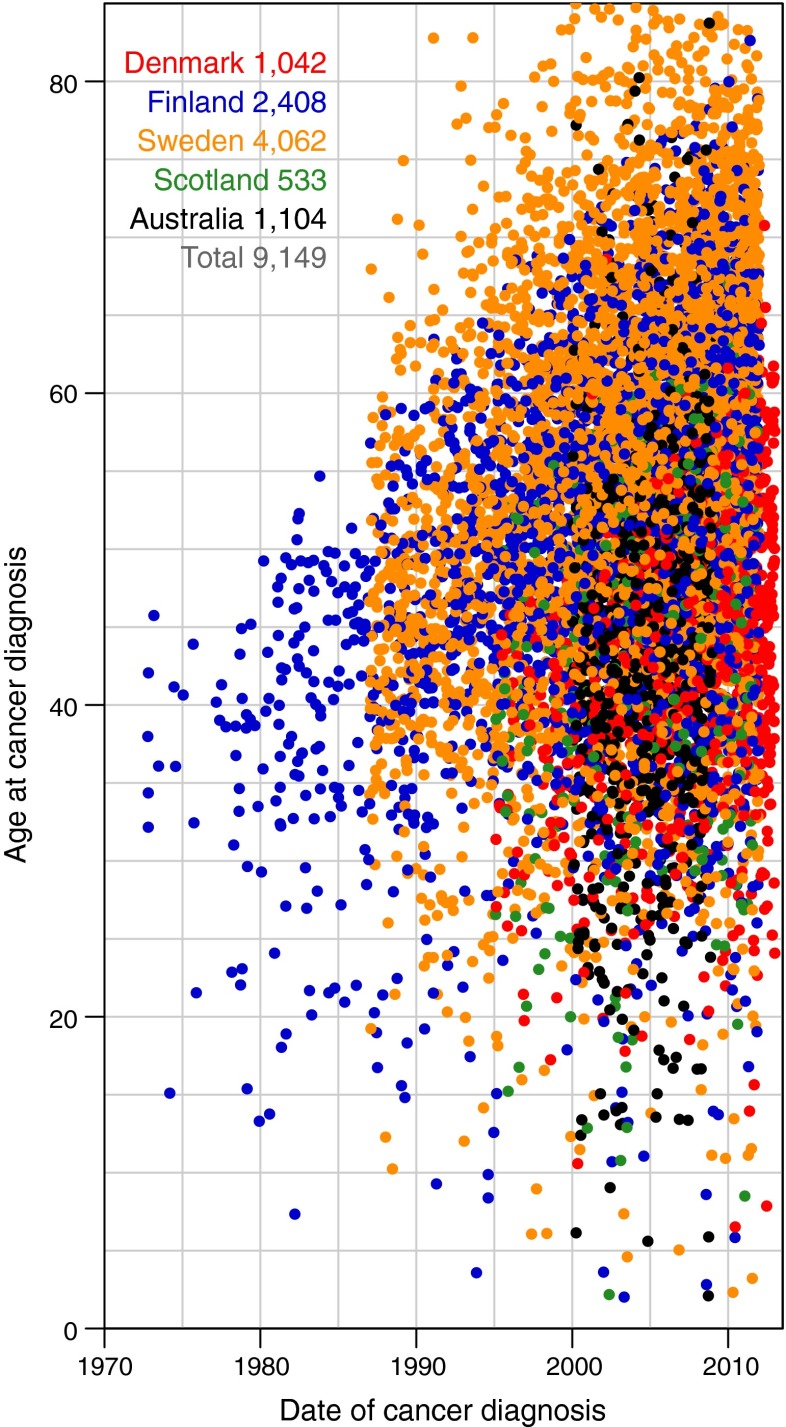
Distribution of cancers by country, age and date of cancer diagnosis

Figure 1 shows the distribution of cancer cases among persons with type 1 diabetes in the five contributing countries. In this study population, the majority of cancer cases occurred between the ages of 40 and 60 years. The median age at cancer diagnosis in this relatively young cohort was 51.1 years (interquartile range 43.5–59.5).

### Basic analyses

**Fig. 2:**
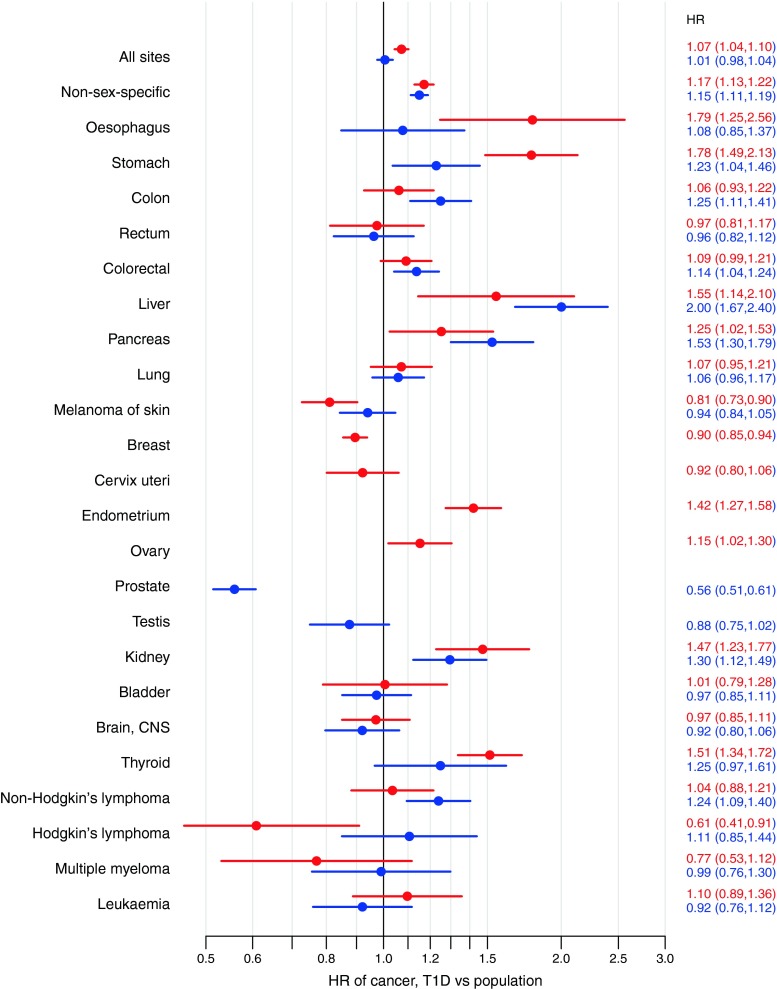
HRs (with 95% CIs) of site-specific cancers associated with type 1 diabetes in men (blue) and women (red) obtained from the analysis of combined country data. HRs are presented on a logarithmic scale. CNS, central nervous system; T1D, type 1 diabetes

We found HRs (95% CIs) for overall cancer of 1.01 (0.98, 1.04) among men and 1.07 (1.04, 1.10) among women (Fig. 2 and ESM Table 2) in comparison with the general population. When analyses were restricted to non-sex-specific cancers (i.e. excluding prostate, testis, breast, cervix, endometrium and ovary cancer), HRs (95% CIs) of 1.15 (1.11, 1.19) among men and 1.17 (1.13, 1.22) among women were observed.

The HRs associated with type 1 diabetes for 23 cancer subsites are shown in Fig. 2. Significantly elevated HRs for both sexes were observed for cancers of the liver, pancreas, kidney and stomach. Women with type 1 diabetes had elevated HRs for cancer of the oesophagus, endometrium, ovary and thyroid. Among men with type 1 diabetes, elevated HRs were observed for colon cancer and non-Hodgkin’s lymphoma. Women with type 1 diabetes exhibited significantly reduced HRs for melanoma, breast cancer and Hodgkin’s lymphoma. Men with type 1 diabetes had a 44% lower incidence of prostate cancer than that observed in the general population. In addition, we found a borderline significant 12% lower risk of testis cancer in men with type 1 diabetes.

There was no significant interaction between age at diagnosis and the HR between type 1 diabetic patients and the general population for any of the five countries.

Significant heterogeneity was observed between HR estimates from the five countries (*p ≤* 0.0001; see ESM Table 2 and ESM Figs 2–4). In particular, there were large differences by country in the HRs for cancer at all sites in men, e.g. type 1 diabetes was associated with an 18% increased cancer incidence in Finland compared with a 10% reduction in Sweden. However, when analyses were performed for cancer subsites by country, there were no consistent differences. Statistically significant heterogeneity in the estimated HRs for cancer by country was found for individual cancer sites, including cancer of the pancreas, kidney, prostate, brain/central nervous system and leukaemia in men and colorectal and cervical cancer in women.

### Analysis by diabetes duration

A total of 7,792 (85.2% of 9,149) patients with type 1 diabetes who had a verifiable date of diabetes diagnosis were subsequently diagnosed with cancer.

**Fig. 3:**
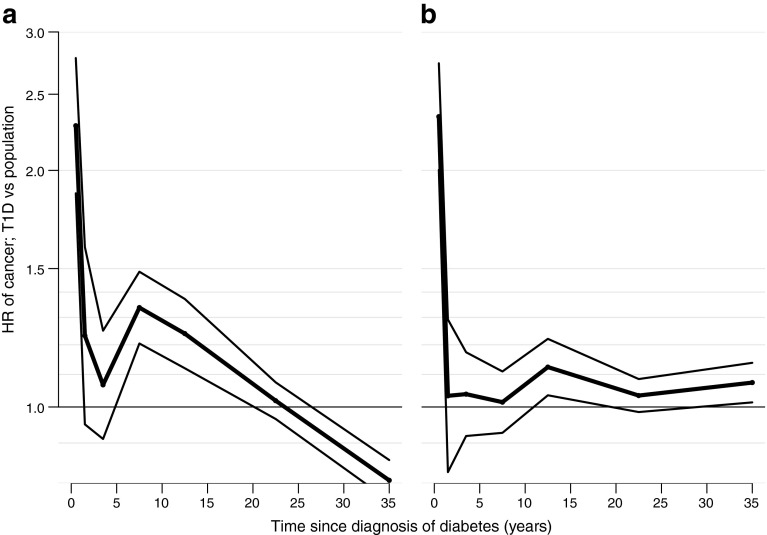
HR (95% CI) of all cancers by duration of diabetes in men (**a**) and women (**b**)

High HRs (95% CIs) for overall cancer occurrence were observed during the first year following the diagnosis of diabetes (men, HR 2.28 [1.87, 2.78]; women, HR 2.34 [2.00, 2.74]; Fig. 3 and ESM Table 3). Following the first year after diabetes diagnosis, the HRs declined to unity (1.03 [0.83, 1.29]) for women and decreased to 1.23 [0.95, 1.60] for men. After around 5 years, the HR of cancer occurrence in men with type 1 diabetes increased to 1.34 [1.20, 1.49]. After 15 years, the cancer incidence among men with type 1 diabetes was similar to that of the general population. The 95% CIs were similar regardless of disease duration because higher incidence rates in people with a longer duration of diabetes resulted in total numbers of events similar to those observed in the larger population with a shorter duration of diabetes.

For most site-specific cancers, the HRs decreased with increasing diabetes duration (ESM Table 4 and ESM Figs 5–8). However, there was very little variation by duration of diabetes for breast cancer incidence, while the HR for endometrial cancer remained elevated for approximately 18 years. The lower incidence of prostate cancer among men with type 1 diabetes became more apparent with increasing duration of diabetes.

## Discussion

In this large multi-country study, we compared the cancer incidence in persons with type 1 diabetes with that observed in the general population, and investigated the influence of diabetes duration on the risk estimates.

We found that women with type 1 diabetes had a marginally elevated incidence of overall cancer compared with the general population. Men with type 1 diabetes did not have an elevated incidence of overall cancer, although the substantial inverse association between type 1 diabetes and prostate cancer incidence in men contributed to the overall result and to these sex differences. When analyses were restricted to non-sex-specific cancers, the excess cancer incidence was similar in both men and women with type 1 diabetes: there were elevated HR estimates of about 15% in the first 20 years after diagnosis. We found that the cancer incidence was substantially higher in both men and women with type 1 diabetes during the first year of follow up compared with a longer duration of diabetes. The cancer incidence subsequently decreased to that of the general population after approximately 20 years of follow up for men and after 5 years of follow up for women.

We found that type 1 diabetes conferred an excess risk of cancer of the stomach, liver, pancreas, endometrium and kidney and a reduced risk of prostate cancer. We also report a lower incidence of breast cancer in women with type 1 diabetes compared with the general population.

There was evidence of heterogeneity in HRs of some cancers by country, though a lack of plausible biological mechanisms leads us to believe these differences are likely to be an artefact.

Previous studies investigating the association between type 1 diabetes status and cancer incidence have reported mixed findings [14], with estimates for the overall cancer risk among persons with type 1 diabetes ranging between 5% lower to 25% higher compared with the general population, although reported 95% CIs include the estimates reported here [10–13, 19]. The discrepancies in these findings may, in part, be due to substantial differences in the criteria used to define type 1 diabetes diagnoses. For example, a UK-based cohort study which identified 214 incident cancers among 23,834 persons with diabetes diagnosed between 1972 and 1986 and reported a standardised incidence ratio of 0.95 (95% CI 0.84, 1.08), used insulin treatment as a proxy for type 1 diabetes [12]. In contrast, in the Swedish cohort study that identified 258 cancer diagnoses among 24,052 persons between 1964 and 2006 and reported an HR of 1.17 (95% CI 1.04, 1.33), ICD codes from hospital admissions plus age were used to ascertain type 1 diabetes status [11]. The authors acknowledged the lack of specific type 1 diabetes diagnostic codes in earlier ICD versions as a possible source of misclassification bias because persons with type 2 diabetes might have been included in the type 1 diabetes study population.

Our finding of a significantly elevated cancer incidence among people with type 1 diabetes in the first year after diagnosis of diabetes may be attributable to ascertainment bias (i.e. earlier detection of pre-existing cancers) and to a lesser extent to reverse causation (i.e. the cancer itself causing diabetes). Previous studies investigating the relationship between type 1 diabetes and cancer have provided only sparse information on the influence of diabetes duration on this association. These studies typically applied wide intervals of diabetes duration in the analyses because of their limited sample sizes. As a consequence, they were unable to perform a detailed analysis of the differences in cancer occurrence according to time since diabetes diagnosis. In one Swedish cohort study including data from 29,187 persons who were hospitalised with type 1 diabetes between 1965 and 1999, HR estimates were stratified within two post-diagnostic periods of 1–14 or ≥15 years following diabetes diagnosis [13]. These analyses yielded standardised incidence ratios of 1.1 (95% CI 1.0, 1.3) and 1.2 (95% CI 1.0, 1.3) for 1–14 and ≥15 years, respectively, which are largely consistent with our results but do not include rates in the first year after diabetes diagnosis.

HRs for site-specific cancers in persons with type 1 diabetes were similar to those observed among persons with all types of diabetes and with type 2 diabetes [1, 20]. This finding suggests a potential common mechanism among persons with type 1 and type 2 diabetes, for example obesity, insulin treatment or hyperglycaemia.

HR estimates for site-specific cancers were highest for cancer types in which obesity is an established risk factor, namely stomach, colorectal, kidney, endometrial and pancreatic cancers [21]. While obesity is less prevalent in persons with type 1 diabetes than in persons with type 2 diabetes, obesity is increasing among persons with type 1 diabetes and may contribute to an increased risk of certain cancers [22, 23]. Further research is therefore required to compare obesity prevalence in people with and without type 1 diabetes.

The influence of glucose-lowering medications on the observed association between diabetes and cancer has been extensively investigated [24, 25]. Our finding of a smaller excess incidence of cancer among persons with type 1 diabetes than was previously observed in persons with type 2 diabetes does not support the notion that insulin therapies contribute to the observed elevated incidence. If exogenous insulin did contribute to the observed association, the strength of the relationship between type 1 diabetes and cancer incidence would be expected to be stronger than that observed among persons with type 2 diabetes because all people with type 1 diabetes are treated with insulin and a minority of people with type 2 diabetes receive insulin treatment. Furthermore, the absence of an association between overall or site-specific cancer risk and increasing duration of diabetes in our study does not support a dose–response relationship between exogenous insulin use and cancer incidence. We did not have sufficiently detailed data to evaluate associations between type 1 diabetes and cancer incidence according to specific types of insulin.

Diabetes-specific metabolic deficiencies such as hyperglycaemia may provide an alternative explanation for the observed excess risk of some cancers among persons with diabetes, given that these deficiencies are common in both type 1 and type 2 diabetes. Hyperglycaemia may be a plausible explanation given the identification of a dose–response relationship between glycated haemoglobin levels and the risk of certain cancers [8, 26, 27]. However, further work is required to elucidate whether obesity or hyperglycaemia in persons with type 1 diabetes is responsible for the observed association with some cancer types.

We report a 10% lower incidence of breast cancer in women with type 1 diabetes compared with the general population. Previous studies assessing the influence of type 2 or unspecified diabetes on breast cancer have displayed a similar or elevated risk compared with the general population, although these findings are largely limited to post-menopausal women [28]. Our contradictory finding may therefore reflect our younger cohort (containing fewer post-menopausal women) or may be confounded by other breast cancer risk factors such as parity.

Our finding of an increased incidence of pancreatic and liver cancer among persons with type 1 diabetes is consistent with some earlier studies. Results from a meta-analysis based on nine studies (but with only a total of 39 cases of pancreatic cancer) indicated that persons with type 1 diabetes were at a twofold increased risk of pancreatic cancer compared with persons without diabetes [29]. A Danish study (distinct from the Danish data in this study) observed an HR of 4.8 (95% CI 2.8, 7.7) of liver cancer among diabetes patients younger than 50 years at diagnosis. Although this definition of type 1 diabetes is not strictly comparable with ours, the result is consistent with our findings [19]. However, the associations seen for pancreatic and liver cancer may be a consequence of reverse causation.

We found an increased risk of thyroid cancer in persons with type 1 diabetes compared with the general population, particularly among women. The worldwide incidence rates of thyroid cancer have increased steeply in recent decades [30, 31], partly due to enhanced detection through the use of more sensitive diagnostic equipment. Consequently, our observation of an increased incidence of thyroid cancer among persons with type 1 diabetes may be a diagnostic artefact because persons with type 1 diabetes receive considerably higher levels of medical attention than those without diabetes.

Although a reduced risk of prostate cancer in persons with type 2 diabetes has been reported in numerous studies [2–5], only one previous study has identified such an association among persons with type 1 diabetes (although some of the latter study data were included in the present study) [10]. The similarity in prostate cancer incidence between type 1 and type 2 diabetic patients suggests a similar underlying mechanism in both types of diabetes. One proposed mechanism involves the lower testosterone levels present in men with diabetes, obesity or both [32]. Higher testosterone levels were previously shown to lead to an increased risk of prostate cancer [33], while hyperglycaemia has also been shown to inhibit testosterone production [34].

### Strengths and limitations

This study used five national cohorts of persons with type 1 diabetes, and is therefore the largest study to date to investigate the relationship between type 1 diabetes and cancer incidence. By combining cases across countries we assembled a sufficiently large sample size to investigate associations with site-specific cancers. The largest previous study included less than one-third of the number of cancers in people with type 1 diabetes compared with our study [14]. The population-based registry approach also minimised selection bias.

A further strength of our study was the inclusion of information on duration of diabetes, which enabled us to assess the influence of ascertainment bias and the potential impact of reverse causation. For example, it is well established that pancreatic cancer can induce diabetes [35].

Possible misclassification of type 2 as type 1 diabetes may have led to an overestimation of the relationship between type 1 diabetes and cancer because of the higher levels of well-established risk factors for cancer (including obesity and smoking) among persons with type 2 diabetes. However, the prevalence of type 2 diabetes below 40 years of age is low and we therefore believe that the influence of misclassification bias is negligible, as shown by our interaction analysis (see http://bendixcarstensen.com/DMCa/T1D/T1D-Ca.pdf (accessed 8 January 2016), section 8.5).

Moreover, we compared cancer incidence rates among persons with type 1 diabetes with those of the general population, instead of only with those of persons with diabetes. This may have diluted the impact of type 1 diabetes on HR estimates, although this effect will be small because of the low prevalence of type 1 diabetes (1.5–4.8 per 1,000 people) in the study populations [36].

In addition, the large number of comparisons performed introduced the potential for chance findings.

Finally, the study population was relatively young. Thus, we were not able to meaningfully evaluate potential changes in cancer incidence among older persons with type 1 diabetes.

### Implications

Our findings do not support changing the policy for cancer screening in persons with type 1 diabetes. Similar recommendations for lifestyle approaches to reduce cancer risk such as weight management, increasing physical activity and avoiding smoking apply to persons with type 1 diabetes as for the general population.

Future work should be directed at ascertaining whether the increased incidence of some cancers among persons with type 1 diabetes leads to a raised risk of cancer mortality among persons with type 1 diabetes.

## Conclusions

We found that, on average, type 1 diabetes confers an excess incidence of several cancers. In particular, persons with type 1 diabetes had a higher incidence of cancer of the liver, pancreas, kidney, endometrium and ovary and a lower incidence of prostate cancer than those in the general population. However, similar to the findings for type 2 diabetes, the HRs of cancer were highest at time of diabetes diagnosis and declined over time.

## Electronic supplementary material

Below is the link to the electronic supplementary material.

ESM (PDF 232 kb)
