# Supplementary material for: Cancer incidence in persons with type 1 diabetes: a five-country study of 9,000 cancers in type 1 diabetic individuals
Source: Diabetologia. 2016 Feb 29;59:980–8. doi: 10.1007/s00125-016-3884-9 (PMC4826427; doi:10.1007/s00125-016-3884-9)
Supplement: Supplementary file 1 — (PDF 232 kb) [file 125_2016_3884_MOESM1_ESM.pdf]

Cancer incidence in type 1 diabetes: A 5-country study of 9,000 cancers in type 1 diabetic individuals

Electronic Supplementary Material

http://BendixCarstensen.com/DMCa/T1D/T1D-CA-Suppl.pdf
February 2016

Compiled Wednesday 10th February, 2016, 21:11
from: /home/bendix/sdc/DMreg/T1DCa/T1D-Ca-Suppl.tex

Bendix Carstensen	Steno Diabetes Center, Gentofte, Denmark
	& Department of Biostatistics, University of Copenhagen
	bxc@steno.dk
	http://BendixCarstensen.com
Stephanie Read	University of Edinburgh, Scotland
Søren Friis	Danish Cancer Society Research Center, Copenhagen, Denmark
Reijo Sund	University of Helsinki, Finland
Ilmo Keskimäki	National Institute for Health and Welfare, Helsinki, Finland
Ann-Marie Svensson	National Swedish Diabetes Register, Gothenburg, Sweden
Rickard Ljung	Karolinska Institutet, Stockholm, Sweden
Sarah Wild	University of Edinburgh, Scotland
Joannes Kerssens	National Records Scotland
Jessica Harding	Baker IDI, Melbourne, Australia
Dianna Magliano	Baker IDI, Melbourne, Australia
Soffia Gudbjörnsdottir	National Swedish Diabetes Register, Gothenburg, Sweden

Contents

1 Introduction **1**

2 Nordic Countries **1**

2.1 Denmark 1

2.2 Finland 1

2.3 Sweden 2

3 Scotland **2**

4 Australia **3**

References **5**

List of Tables

1 Person-years and number of cancer cases in T1D patients (0–85 years) by sex, country and site. "All sites" comprise more that the single sites together, as other and unspecified sites are included. 6

2 HR of cancer among T1D patients relative to the general population by sex and country and P-value for homogeneity across countries. The non-sex-specific sites are all sites except breast, cervix, endometrium, ovary, prostate and testis. 7

3 HR of cancer among T1D patients relative to the general population for all sites and non sex-specific sites by sex and duration of diabetes. The non-sex-specific sites are all sites except breast, cervix, endometrium, ovary, prostate and testis. 8

4 HR of cancer among T1D patients relative to the general population by site, sex and duration of diabetes. 9

List of Figures

1 Distribution of cancer events (top half) and person-years (bottom half) in T1D patients by age and calendar time for all (first two columns), and by age, calendar time and diabetes duration for those with duration of DM known (last three columns). 10

2 HR of different cancers from the joint analysis (thick, full color), and the interaction with country (thin, pale color, ordered bottom-up as: DK, FI, SE, SC, AU). Blue: men, red: women. 11

3 Hazard ratio of all cancers between T1D patients and the general population, by country 12

4 Hazard ratio of non-sex-specific cancers between T1D patients and the general population, by country 12

5 Hazard ratio of cancer of select sites between T1D patients and the general population. Pooled analysis. 13

6 Hazard ratio of cancer of select sites between T1D patients and the general population. Pooled analysis. . . . 14
7 Hazard ratio of cancer of select sites between T1D patients and the general population. Pooled analysis. . . . 15
8 Hazard ratio of cancer of select sites between T1D patients and the general population. Pooled analysis. . . . 16

1 Introduction

Details of each country's source of diabetes and cancer registrations follow below.

2 Nordic Countries

Population-level data on cancer incidence for Denmark, Sweden and Finland were extracted from NORDCAN [1], a multinational registry of cancer cases in each of the Nordic countries (Denmark, Sweden, Finland, Iceland, Norway and the Faroe Islands). This registry was formed in 2003 and is populated by data from each country's cancer registry.

2.1 Denmark

The Danish National Diabetes Register was established in 2006 [2] and was based on three existing health care registers, the Danish Civil Registration System, the National Patient Register and the National Health Service Register. As of January 1995, the Danish National Diabetes Register was considered complete with regards to incident cases. The Danish Cancer Registry [3] was established in 1943 and cancer reporting to this registry has been mandatory since 1987. A system of multiple notifications from several data sources including the Danish Pathology Register has ensured that complete coverage of cancer diagnoses has been attained. Data from these registers were linked using the Central Person Register number, a unique patient identifier assigned to every person in Denmark as of 1968 [4]. The period of follow-up was January 1995 until December 2012, a total of 18 years. In addition to patients diagnosed with type 1 diabetes after 1995, prevalent cases who were diagnosed before 1995 but were under 40 years of age in 1995, and therefore diagnosed below 40 years of age were also included in this study.

No ethical approval is needed for register based studies in Denmark. Approval for linkage was obtained from the Danish Data Protection Board.

2.2 Finland

Data on diabetes diagnoses were obtained from FinDM, a research database maintained by the National Institute for Health and Welfare (THL) [5]. This database contains all people identified to have a diabetes record since 1964 in a number of health care registries including hospital admissions and prescription databases. These nationwide health care registers are held by the National Institute for Health and Welfare (THL), the Social Insurance Institution of Finland (KELA) and Statistics Finland. A comparison against a local diabetes register of the Helsinki metropolitan area has demonstrated good coverage of diabetic patients in the nationwide database [6]. Follow-up data were obtained from the registers using the Finnish personal identification codes. In the present study, those who were not permanent residents of Finland or had gestational diabetes only were excluded from the analyses, and the type of diabetes was determined according to the study criteria. The Finnish Cancer Registry was launched in 1953 and is a national database of all cancer cases in Finland [7]. The National Board of Health established compulsory reporting of all diagnosed or suspected cases of cancer to the registry in 1961. The completeness of solid tumour registrations was reported to be over 99% in 1994 [8] and 96% of registered

diagnoses were valid between 1985 and 1997 [9]. The period of follow-up in Finland was from 1972 until 2010.

Permissions to use the data for the study were received from all respective competent register authorities. The linkages were performed by competent authorities and the research group received anonymized data. Ethical approval for the study for received from the Research Ethics Committee of the Helsinki and Uusimaa hospital district.

2.3 Sweden

Launched as a quality assurance register in 1996, the Swedish National Diabetes Register includes patient demographic and clinical data such as treatments, complications and risk factor levels [10]. The registry is populated by data uploaded via clinical record databases by trained physicians and nurses following patient appointments at hospital outpatient clinics or primary health care centers. To ascertain the presence of and the date of diabetes diagnosis, both the Swedish National Diabetes register and the National Patient Register were searched for earliest mention of diabetes. For patients with two distinct dates of first diabetes mention, the earliest date was used as the date of diagnosis. The Swedish Cancer Register was established in 1958 and includes data on every primary malignancy, certain benign tumours and precancerous lesions in Sweden. Every clinician, pathologist and cytologist in Sweden must notify the National Board of Health and Welfare of each person who has been diagnosed with a new primary malignancy. In a comparison with the National Patient Register which comprises of information on all in-hospital care and outpatient specialist care in Sweden, the rate of non-reporting to the National Cancer Registry was estimated to be 3.7% in 1998 [11]. Data linkage between registries was achieved using the personal identification number, a unique patient number assigned to each Swedish citizen. For this study, patients diagnosed with type 1 diabetes between 1987 and 2011 were included.

This study was approved by the Central Ethical Review Board in Gothenburg.

3 Scotland

To determine patient diabetes status, the use of ICD-9 codes 250 and ICD-10 codes E10-E14 to identify diabetes diagnoses in Scottish General Acute/Inpatient (SMR-01) dataset was used because the national diabetes register was not complete until 2004. This dataset was established in 1960 and captures hospital admission data, including the primary medical condition which led to hospital admission and up to five other co-existing conditions, from all hospitals in Scotland [12]. A validation study using data from the national diabetes register after 2004 estimated that the SMR-01 captures approximately 72% of diabetes diagnoses in people aged below 30 [13].

The Scottish Cancer registry (Scottish Morbidity Record SMR-06) was established in 1958 and has been managed by the Information Services Division (ISD) of National Services Scotland since 1997 [14]. SMR-06 captures cancer registration data for the entire Scottish population, with approximately 47,000 cancer registrations made annually. Data on cancer diagnosis dates and cancer sites occurring between 1995 and 2011 were retrieved from SMR-06. To ascertain date and cause of death, data from the General Register Office

of Scotland death records (SMR99) were retrieved. Data linkage between these registers was achieved using the Community Health Index, a unique patient identification number and probabilistic linkage. The study period for Scotland was from 1995 until 2011.

Approval for the creation and analysis of the Scottish linked dataset containing no personal identifying information was obtained from the Scottish Care Information - Diabetes Collaboration (SCI-DC) steering committee, the Scottish multi-centre research ethics committee (reference number 11/AL/0225), the Privacy Advisory Committee of National Health Service National Services Scotland and Caldicott guardians.

4 Australia

The National Diabetes Services Scheme was established in 1987 and currently captures data for approximately 80 to 90% of all people with a diagnosis of diabetes in Australia [15]. This registry was formed to provide subsidized diabetes products and information to people with diabetes in Australia. Registration to NDSS is free and is certified by medical practitioners. Prior to 2000, completeness of the diagnosis date variable was low and therefore a number of persons diagnosed with diabetes aged under 40 years during this time could not be included in these analyses [16].

We included type 1 diabetes who were registered on the NDSS in the years 2000 to 2008 (including all those registered before 2000 still alive on January 1 2000). The year 2000 was chosen as the start date as this time followed a unification of state-based registries as well as an improvement in data quality, including better capture of diabetes diagnosis date.

Type 1 diabetes status was assigned to registrants who satisfied all of the following conditions:

- recorded as type 1 on the NDSS registry
- diagnosed before 40 years of age
- were taking insulin

Registration date was used as a proxy for diagnosis date, as a large proportion of type 1 registrants (54%) had missing date of diagnosis, many of whom registered in the early years of the operation of the NDSS and had had diabetes for a number of years. We chose 40 years as the cut-off age to minimize the number of people with type 1 diabetes that we would miss, without misclassifying significant numbers of people with type 2 as type 1 [17]. Additionally, registrants who were recorded as type 2 on the registry, but diagnosed before age 30 and were taking insulin within 1 year of diagnosis date were reclassified as having type 1 diabetes.

The Australian Cancer Database was launched in 1982 and is a register of all primary, malignant cancers in Australia, where notification of cancer diagnoses is mandatory. The registry currently receives notifications on cancer diagnoses from several sources including hospitals, pathology laboratories, radiotherapy centers and death registries. Data linkage of these registries was completed by the Australian Institute of Health and Welfare using the Fellegi and Sunter framework [18]. This framework uses names first, second, third), sex and date of birth to conduct the linkage. Incident cases of cancer occurring between 1999 and 2009 retrieved from the website of the Australian Institute of Health and Welfare, at

https://www.aihw.gov.au/acim-books/ (accessed March 2014); we used this slightly longer period for the population rates to get more stable estimates of the population rates in the period 2000–2008.

This study was approved by the Alfred Health Human Ethics Committee and the Australian Institute of Health and Welfare Ethics Committee.

References

[1] G. Engholm, J. Ferlay, N. Christensen, F. Bray, M. L. Gjerstorff, A. Klint, J. E. Køtlum, E. Olafsdottir, E. Pukkala, and H. H. Storm. NORDCAN–a Nordic tool for cancer information, planning, quality control and research. *Acta Oncol*, 49(5):725–736, 2010.

[2] B. Carstensen, Christensen J.K., Marcussen M.M., and Borch-Johnsen K. The National Diabetes Register. *Scandinavian Journal of Public Health*, 39(7 suppl):58–61, 2011.

[3] M. L. Gjerstorff. The Danish Cancer Registry. *Scand J Public Health*, 39(7 Suppl):42–45, July 2011.

[4] Pedersen C.B. The Danish civil registration system. *Scand J Public Health*, 39 (7 suppl):22–25, July 2011.

[5] Sund R and Koski S. FinDM II: On the register-based measurement of the prevalence and incidence of diabetes and its long-term complications. Technical report, Finnish National Public Health Institute, 2009.

[6] Sund R, Harno K, Ranta S, and Tolppanen EM. Evaluation of case inclusion in two population-based diabetes registers. *Finnish J eHealth eWelfare*, 2:136–46, 2010.

[7] Pukkala E and Rautalahti M. Cancer in Finland: Publications from the Cancer Society of Finland. Technical report, Cancer Society of Finland, 2013.

[8] L. Teppo, E. Pukkala, and M. Lehtonen. Data quality and quality control of a population-based cancer registry. Experience in Finland. *Acta Oncol*, 33(4):365–369, 1994.

[9] P. Korhonen, N. Malila, E. Pukkala, L. Teppo, D. Albanes, and J. Virtamo. The Finnish Cancer Registry as follow-up source of a large trial cohort–accuracy and delay. *Acta Oncol*, 41(4):381–388, 2002.

[10] S. Gudbjörnsdottir, J. Cederholm, P. M. Nilsson, and B. Eliasson. The National Diabetes Register in Sweden: an implementation of the St. Vincent Declaration for Quality Improvement in Diabetes Care. *Diabetes Care*, 26(4):1270–1276, Apr 2003.

[11] L. Barlow, K. Westergren, L. Holmberg, and M. Talback. The completeness of the Swedish Cancer Register: a sample survey for year 1998. *Acta Oncol*, 48(1):27–33, 2009.

[12] S. J. Livingstone, H. C. Looker, E. J. Hothersall, S. H. Wild, R. S. Lindsay, J. Chalmers, S. Cleland, G. P. Leese, J. McKnight, A. D. Morris, D. W. Pearson, N. R. Peden, J. R. Petrie, S. Philip, N. Sattar, F. Sullivan, and H. M. Colhoun. Risk of cardiovascular disease and total mortality in adults with type 1 diabetes: Scottish registry linkage study. *PLoS Med.*, 9(10):e1001321, 2012.

[13] Varnum CA. A validation study to assess the use of hospital records as the primary means of identifying individuals with type 1 diabetes. Technical report, Unpublished.

[14] Harris V, Sandridge AL, Black RJ, Brewster DH, and Gould A. Cancer registration statistics Scotland 1986-1995. Technical report, Edinburgh, 1998.

[15] Diabetes Australia. NDSS Registrant Details Audit Summary Report. Technical report, 2015.

[16] Harding JL, Shaw JE, Peeters A, Guiver T, Davidson S, and Magliano DJ. Mortality trends among people with type 1 and type 2 diabetes in australia: 1997-2010. *Diabetes Care*, 37:2579–2586, 2014.

[17] Kenny SJ, Aubert RE, and Linda SG. Prevalence and incidence of non-insulin-dependent diabetes. Technical report, U.S. Government. Printing Office, 1995.

[18] IP Fellegi and AB Sunter. A theory for record linkage. *Journal of the American Statistical Association*, 64:1183–1210, 1969.

Table 1: *Person-years and number of cancer cases in T1D patients (0–85 years) by sex, country and site. "All sites" comprise more that the single sites together, as other and unspecified sites are included.*

	Men						Women						
Cancer site	DK	FI	SE	SC	AU	Sum	DK	FI	SE	SC	AU	Sum	Total
Person-years (1000)[1]	255.6	547.9	737.5	178.7	255.5	1,975.1	289.0	636.8	631.1	145.5	255.4	1,957.7	3,932.9
All sites	401	1,000	1,882	253	504	4,040	641	1,408	2,180	280	600	5,109	9,149
Non sex-specific	352	832	1,480	222	443	3,329	351	680	1,122	146	364	2,663	5,992
Person-years (1000s)[2]	258.9	553.8	746.0	179.5	255.5	1,993.7	293.9	648.0	645.2	146.8	255.4	1,989.3	3,983.0
Oesophagus	4	16	29	9	9	67	1	5	14	7	3	30	97
Stomach	6	47	64	5	12	134	14	50	39	4	13	120	254
Colon	26	56	173	18		273	16	66	119	9		210	483
Rectum	13	46	84	15		158	10	41	57	6		114	272
Colorectal	39	102	257	33	61	492	26	107	176	15	60	384	876
Liver	9	34	44	12	14	113	6	8	17	3	7	41	154
Pancreas	15	54	52	7	19	147	9	41	30	5	8	93	240
Lung	39	119	134	37	41	370	38	58	128	19	29	272	642
Melanoma of skin	21	60	122	16	86	305	59	59	103	15	72	308	613
Breast							184	546	710	99	184	1,723	1,723
Cervix uteri							48	36	85	14	11	194	194
Corpus uteri							40	97	149	12	25	323	323
Ovary							25	80	114	11	22	252	252
Prostate	12	148	341	11	41	553							553
Testis	37	23	57	20	22	159							159
Kidney	23	67	62	14	21	187	15	47	37	4	15	118	305
Bladder	17	49	124	11	12	213	7	16	35	6	2	66	279
Brain, CNS	31	35	79	18	17	180	42	32	98	28	13	213	393
Thyroid	6	18	15	4	15	58	29	104	51	12	45	241	299
Non-Hodgkin's lymphoma	26	56	111	21	30	244	14	46	56	8	19	143	387
Hodgkin's lymphoma	11	14	20		11	56	4	9	9		2	24	80
Multiple myeloma	4	14	26	3	6	53	5	8	13	0	2	28	81
Leukaemia	14	39	38	13		104	16	33	28	9		86	190

[1] Follow-up only to the first primary tumour of any kind or end of study. [2] Follow-up till death or end of study; used for analysis of specific sites.

Table 2: *HR of cancer among T1D patients relative to the general population by sex and country and P-value for homogeneity across countries. The non-sex-specific sites are all sites except breast, cervix, endometrium, ovary, prostate and testis.*

Cancer site	Sex	Denmark	Finland	Sweden	Scotland	Australia	Joint	P
All sites	M	1.10 (1.00,1.22)	1.18 (1.11,1.25)	0.90 (0.86,0.94)	1.06 (0.94,1.20)	1.06 (0.98,1.15)	1.01 (0.98,1.04)	0.000
	W	1.00 (0.93,1.08)	1.00 (0.95,1.06)	1.13 (1.08,1.18)	1.05 (0.94,1.19)	1.13 (1.05,1.21)	1.07 (1.04,1.10)	0.003
Non-sex-specific	M	1.20 (1.08,1.33)	1.30 (1.21,1.39)	1.06 (1.00,1.11)	1.14 (1.00,1.30)	1.21 (1.11,1.31)	1.15 (1.11,1.19)	0.000
	W	1.16 (1.05,1.29)	1.08 (1.00,1.16)	1.18 (1.11,1.25)	1.26 (1.07,1.48)	1.31 (1.20,1.44)	1.17 (1.13,1.22)	0.023
Oesophagus	M	0.58 (0.22,1.56)	1.33 (0.81,2.18)	1.04 (0.72,1.49)	1.07 (0.56,2.06)	1.30 (0.68,2.50)	1.08 (0.85,1.37)	0.581
	W	0.42 (0.06,3.01)	1.20 (0.50,2.90)	2.01 (1.19,3.41)	3.86 (1.83,8.14)	1.96 (0.63,6.09)	1.79 (1.25,2.56)	0.097
Stomach	M	0.72 (0.32,1.60)	1.49 (1.11,1.98)	1.21 (0.95,1.55)	0.85 (0.35,2.06)	1.15 (0.65,2.03)	1.23 (1.04,1.46)	0.347
	W	2.65 (1.55,4.51)	1.84 (1.39,2.43)	1.47 (1.07,2.02)	1.51 (0.56,4.04)	2.28 (1.32,3.93)	1.78 (1.49,2.13)	0.373
Colon	M	1.30 (0.88,1.92)	1.23 (0.95,1.60)	1.26 (1.08,1.46)	1.19 (0.75,1.88)		1.25 (1.11,1.41)	0.990
	W	0.73 (0.45,1.19)	1.14 (0.90,1.46)	1.10 (0.92,1.32)	0.86 (0.45,1.66)		1.06 (0.93,1.22)	0.326
Rectum	M	0.90 (0.52,1.55)	1.31 (0.98,1.75)	0.83 (0.67,1.02)	1.19 (0.71,1.97)		0.96 (0.82,1.12)	0.083
	W	0.82 (0.44,1.53)	1.17 (0.86,1.59)	0.91 (0.71,1.18)	0.85 (0.38,1.90)		0.97 (0.81,1.17)	0.590
Colorectal	M	1.13 (0.83,1.55)	1.26 (1.04,1.54)	1.07 (0.95,1.21)	1.20 (0.85,1.68)	1.20 (0.95,1.52)	1.14 (1.04,1.24)	0.681
	W	0.76 (0.52,1.12)	1.15 (0.95,1.40)	1.03 (0.89,1.20)	0.86 (0.52,1.44)	1.54 (1.21,1.95)	1.09 (0.99,1.21)	0.013
Liver	M	2.12 (1.10,4.11)	2.64 (1.88,3.71)	1.45 (1.07,1.95)	3.49 (1.97,6.17)	2.35 (1.52,3.66)	2.00 (1.67,2.40)	0.026
	W	2.52 (1.12,5.69)	1.05 (0.52,2.11)	1.23 (0.77,1.99)	2.89 (0.93,9.04)	3.51 (1.75,7.05)	1.55 (1.14,2.10)	0.073
Pancreas	M	1.84 (1.10,3.07)	1.89 (1.44,2.47)	1.08 (0.82,1.42)	1.50 (0.71,3.15)	2.55 (1.68,3.88)	1.53 (1.30,1.79)	0.006
	W	1.37 (0.71,2.65)	1.58 (1.16,2.16)	0.84 (0.59,1.20)	1.95 (0.81,4.71)	1.96 (1.05,3.66)	1.25 (1.02,1.53)	0.038
Lung	M	1.27 (0.92,1.74)	1.22 (1.02,1.46)	0.79 (0.67,0.94)	1.42 (1.02,1.96)	1.45 (1.10,1.92)	1.06 (0.96,1.17)	0.000
	W	1.00 (0.73,1.38)	1.15 (0.89,1.49)	1.01 (0.85,1.20)	1.14 (0.73,1.79)	1.32 (0.92,1.89)	1.07 (0.95,1.21)	0.698
Melanoma	M	0.50 (0.32,0.76)	1.16 (0.90,1.50)	0.92 (0.77,1.10)	0.94 (0.57,1.54)	1.03 (0.86,1.25)	0.94 (0.84,1.05)	0.007
	W	0.69 (0.53,0.89)	0.86 (0.66,1.11)	0.80 (0.66,0.97)	0.69 (0.42,1.15)	0.91 (0.75,1.12)	0.81 (0.73,0.90)	0.488
Breast	W	0.74 (0.64,0.86)	0.88 (0.81,0.96)	0.95 (0.89,1.03)	0.91 (0.75,1.11)	0.90 (0.79,1.03)	0.90 (0.85,0.94)	0.048
Cervix uteri	W	0.85 (0.64,1.13)	0.99 (0.72,1.38)	1.13 (0.91,1.40)	0.71 (0.42,1.20)	0.54 (0.32,0.90)	0.92 (0.80,1.06)	0.043
Endometrium	W	2.25 (1.65,3.09)	1.28 (1.05,1.57)	1.37 (1.16,1.60)	1.42 (0.80,2.50)	1.49 (1.05,2.12)	1.42 (1.27,1.58)	0.075
Ovary	W	1.02 (0.69,1.51)	1.16 (0.93,1.45)	1.19 (0.99,1.44)	0.75 (0.41,1.35)	1.40 (0.95,2.08)	1.15 (1.02,1.30)	0.433
Prostate	M	0.53 (0.30,0.93)	0.79 (0.67,0.92)	0.51 (0.46,0.56)	0.53 (0.29,0.96)	0.48 (0.36,0.65)	0.56 (0.51,0.61)	0.001
Testis	M	0.78 (0.56,1.07)	0.89 (0.59,1.34)	0.98 (0.76,1.28)	0.88 (0.56,1.36)	0.82 (0.55,1.21)	0.88 (0.75,1.02)	0.844
Kidney	M	1.65 (1.09,2.50)	1.75 (1.37,2.22)	0.96 (0.75,1.23)	1.49 (0.88,2.51)	1.17 (0.79,1.75)	1.30 (1.12,1.49)	0.010
	W	2.27 (1.36,3.80)	1.68 (1.26,2.24)	1.17 (0.85,1.61)	1.01 (0.38,2.71)	1.52 (0.92,2.53)	1.47 (1.23,1.77)	0.206
Bladder	M	0.94 (0.58,1.51)	1.34 (1.01,1.77)	0.87 (0.73,1.04)	0.84 (0.46,1.51)	1.37 (0.78,2.42)	0.97 (0.85,1.11)	0.112
	W	0.99 (0.47,2.09)	1.34 (0.82,2.20)	0.87 (0.62,1.21)	1.54 (0.69,3.45)	0.82 (0.20,3.27)	1.01 (0.79,1.28)	0.552
Brain, CNS	M	1.15 (0.81,1.64)	0.69 (0.50,0.96)	0.86 (0.69,1.08)	1.05 (0.66,1.68)	1.44 (0.96,2.17)	0.92 (0.80,1.06)	0.057
	W	1.49 (1.10,2.02)	0.38 (0.27,0.54)	1.09 (0.90,1.33)	1.60 (1.10,2.32)	2.23 (1.48,3.36)	0.97 (0.85,1.11)	0.000
Thyroid	M	1.14 (0.51,2.56)	1.28 (0.80,2.03)	1.01 (0.61,1.69)	1.53 (0.57,4.11)	1.52 (0.93,2.48)	1.25 (0.97,1.61)	0.833
	W	1.64 (1.13,2.36)	1.54 (1.27,1.87)	1.45 (1.10,1.91)	1.77 (1.00,3.13)	1.43 (1.09,1.86)	1.51 (1.34,1.72)	0.941
Non-Hodgkin lymphoma	M	1.32 (0.89,1.94)	1.08 (0.83,1.41)	1.29 (1.07,1.55)	1.48 (0.96,2.27)	1.21 (0.88,1.66)	1.24 (1.09,1.40)	0.754
	W	0.93 (0.55,1.57)	0.92 (0.69,1.23)	1.07 (0.82,1.39)	1.02 (0.51,2.05)	1.32 (0.90,1.92)	1.04 (0.88,1.21)	0.675
Hodgkin lymphoma	M	1.42 (0.78,2.58)	0.83 (0.49,1.40)	1.17 (0.75,1.82)		1.24 (0.69,2.25)	1.11 (0.85,1.44)	0.543
	W	0.59 (0.22,1.57)	0.63 (0.33,1.22)	0.82 (0.43,1.58)		0.27 (0.07,1.08)	0.61 (0.41,0.91)	0.471
Multiple myeloma	M	0.99 (0.37,2.65)	1.35 (0.80,2.29)	0.86 (0.58,1.26)	1.01 (0.33,3.15)	1.03 (0.46,2.29)	0.99 (0.76,1.30)	0.777
	W	1.50 (0.62,3.63)	0.79 (0.39,1.58)	0.72 (0.42,1.25)	0.00 (0.00, inf)	0.56 (0.14,2.26)	0.77 (0.53,1.12)	0.532
Leukaemia	M	1.02 (0.60,1.73)	1.39 (1.01,1.91)	0.61 (0.44,0.84)	1.47 (0.85,2.53)		0.92 (0.76,1.12)	0.001
	W	1.43 (0.87,2.35)	1.30 (0.92,1.83)	0.76 (0.53,1.10)	1.81 (0.94,3.50)		1.10 (0.89,1.36)	0.046

Table 3: *HR of cancer among T1D patients relative to the general population for all sites and non sex-specific sites by sex and duration of diabetes. The non-sex-specific sites are all sites except breast, cervix, endometrium, ovary, prostate and testis.*

Sex	Duration	All sites	Non sex-specific
M	0–1	2.28 (1.87,2.78)	2.81 (2.30, 3.43)
	1–2	1.23 (0.95,1.60)	1.26 (0.95, 1.68)
	2–5	1.07 (0.91,1.25)	1.11 (0.93, 1.31)
	5–10	1.34 (1.20,1.49)	1.44 (1.29, 1.61)
	10–15	1.24 (1.12,1.37)	1.30 (1.16, 1.44)
	15–30	1.02 (0.97,1.07)	1.16 (1.09, 1.23)
	30+	0.81 (0.76,0.86)	0.85 (0.79, 0.91)
W	0–1	2.34 (2.00,2.74)	3.01 (2.51, 3.61)
	1–2	1.03 (0.83,1.29)	1.28 (0.98, 1.67)
	2–5	1.04 (0.92,1.17)	1.07 (0.90, 1.26)
	5–10	1.01 (0.93,1.11)	1.12 (0.99, 1.27)
	10–15	1.12 (1.03,1.22)	1.22 (1.09, 1.38)
	15–30	1.03 (0.99,1.09)	1.11 (1.04, 1.19)
	30+	1.07 (1.01,1.14)	0.94 (0.87, 1.02)

Table 4: *HR of cancer among T1D patients relative to the general population by site, sex and duration of diabetes.*

Sex	Duration	Colorectal	Lung	Melanoma	Thyroid	Kidney
M	0–1	3.85 (2.00, 7.41)	0.99 (0.14, 7.00)	1.08 (0.48, 2.40)	3.56 (1.33, 9.48)	3.65 (1.37, 9.75)
	1–2	1.12 (0.36, 3.47)	— (—, —)	0.65 (0.24, 1.73)	0.83 (0.12, 5.89)	2.42 (0.78, 7.51)
	2–5	1.36 (0.79, 2.34)	0.98 (0.41, 2.36)	0.51 (0.28, 0.95)	1.35 (0.56, 3.26)	0.91 (0.34, 2.43)
	5–10	1.60 (1.15, 2.23)	2.02 (1.41, 2.90)	0.93 (0.65, 1.32)	1.82 (1.01, 3.29)	2.07 (1.35, 3.18)
	10–15	1.11 (0.79, 1.56)	1.43 (1.03, 1.99)	1.04 (0.74, 1.47)	1.31 (0.63, 2.76)	2.09 (1.44, 3.03)
	15–30	1.14 (0.98, 1.33)	1.20 (1.02, 1.40)	1.01 (0.82, 1.25)	0.96 (0.54, 1.69)	1.40 (1.11, 1.77)
	30+	1.00 (0.86, 1.16)	0.65 (0.53, 0.81)	0.93 (0.72, 1.20)	0.77 (0.32, 1.85)	0.71 (0.49, 1.03)
W	0–1	2.82 (1.34, 5.93)	4.59 (1.72,12.24)	0.63 (0.28, 1.40)	3.29 (2.01, 5.38)	2.82 (0.70,11.27)
	1–2	0.71 (0.18, 2.85)	0.94 (0.13, 6.68)	0.58 (0.26, 1.29)	1.77 (0.92, 3.41)	2.60 (0.65,10.39)
	2–5	0.70 (0.33, 1.47)	0.69 (0.22, 2.14)	0.71 (0.47, 1.07)	1.30 (0.84, 2.02)	3.11 (1.55, 6.24)
	5–10	0.94 (0.62, 1.45)	0.81 (0.43, 1.50)	0.63 (0.45, 0.89)	1.73 (1.28, 2.34)	1.67 (0.87, 3.22)
	10–15	1.19 (0.84, 1.67)	0.53 (0.28, 0.98)	0.97 (0.71, 1.31)	1.85 (1.34, 2.56)	2.68 (1.68, 4.25)
	15–30	1.15 (0.97, 1.36)	1.26 (1.04, 1.51)	0.68 (0.53, 0.87)	1.34 (1.03, 1.74)	1.65 (1.24, 2.20)
	30+	0.95 (0.79, 1.14)	0.98 (0.79, 1.22)	0.98 (0.74, 1.28)	1.29 (0.83, 2.00)	0.72 (0.45, 1.16)
		Bladder	Brain, CNS	Non-Hodgkin	Liver	Pancreas
M	0–1	3.98 (1.49,10.63)	2.63 (1.55, 4.44)	3.60 (1.99, 6.50)	6.39 (1.59,25.60)	33.80 (19.50,58.57)
	1–2	1.72 (0.43, 6.87)	0.90 (0.37, 2.16)	1.81 (0.81, 4.03)	2.92 (0.41,20.78)	6.43 (2.07,19.99)
	2–5	0.96 (0.36, 2.57)	0.74 (0.42, 1.31)	1.26 (0.73, 2.17)	0.87 (0.12, 6.16)	3.81 (1.81, 8.01)
	5–10	1.62 (0.99, 2.64)	0.97 (0.65, 1.43)	1.20 (0.79, 1.82)	3.33 (1.73, 6.42)	2.86 (1.69, 4.84)
	10–15	1.16 (0.72, 1.87)	0.93 (0.62, 1.41)	1.49 (1.04, 2.15)	3.23 (1.87, 5.57)	0.67 (0.28, 1.61)
	15–30	1.12 (0.90, 1.39)	0.73 (0.55, 0.98)	1.05 (0.82, 1.34)	2.51 (1.90, 3.31)	1.68 (1.31, 2.17)
	30+	0.76 (0.60, 0.96)	0.77 (0.53, 1.12)	1.20 (0.93, 1.55)	1.00 (0.65, 1.53)	0.83 (0.58, 1.19)
W	0–1	8.39 (2.70,26.14)	2.84 (1.77, 4.57)	1.48 (0.48, 4.59)	— (—, —)	29.53 (15.22,57.28)
	1–2	— (—, —)	2.07 (1.20, 3.57)	— (—, —)	— (—, —)	— (—, —)
	2–5	1.38 (0.35, 5.54)	0.75 (0.45, 1.27)	1.45 (0.78, 2.70)	4.91 (1.83,13.14)	1.47 (0.37, 5.88)
	5–10	2.77 (1.44, 5.34)	1.11 (0.79, 1.57)	0.80 (0.43, 1.49)	1.84 (0.59, 5.71)	1.72 (0.77, 3.83)
	10–15	0.89 (0.33, 2.37)	0.78 (0.52, 1.19)	1.65 (1.09, 2.51)	3.48 (1.65, 7.32)	1.94 (1.04, 3.61)
	15–30	0.89 (0.58, 1.38)	0.77 (0.60, 0.99)	1.02 (0.77, 1.35)	1.44 (0.84, 2.49)	1.12 (0.79, 1.59)
	30+	1.00 (0.68, 1.47)	0.77 (0.54, 1.09)	0.76 (0.52, 1.10)	0.93 (0.48, 1.79)	0.87 (0.59, 1.29)
		Prostate	Breast	Endometrium	Cervix	Ovary
M/W	0–1	— (—, —)	0.89 (0.54, 1.45)	13.67 (7.54,24.78)	1.19 (0.62, 2.29)	1.89 (0.79, 4.54)
	1–2	— (—, —)	0.84 (0.53, 1.33)	2.11 (0.53, 8.45)	0.50 (0.19, 1.32)	0.67 (0.17, 2.69)
	2–5	1.00 (0.14, 7.11)	0.83 (0.66, 1.06)	4.03 (2.43, 6.71)	0.80 (0.51, 1.26)	1.98 (1.28, 3.07)
	5–10	0.76 (0.32, 1.82)	0.72 (0.61, 0.86)	2.85 (1.98, 4.10)	1.08 (0.78, 1.50)	1.35 (0.93, 1.96)
	10–15	0.65 (0.39, 1.07)	0.87 (0.76, 1.01)	2.20 (1.59, 3.04)	1.16 (0.81, 1.66)	1.39 (0.99, 1.94)
	15–30	0.52 (0.45, 0.60)	0.87 (0.81, 0.95)	1.05 (0.86, 1.28)	1.04 (0.79, 1.37)	1.03 (0.83, 1.27)
	30+	0.59 (0.53, 0.66)	1.08 (0.98, 1.19)	1.17 (0.95, 1.43)	0.67 (0.39, 1.15)	1.05 (0.80, 1.38)

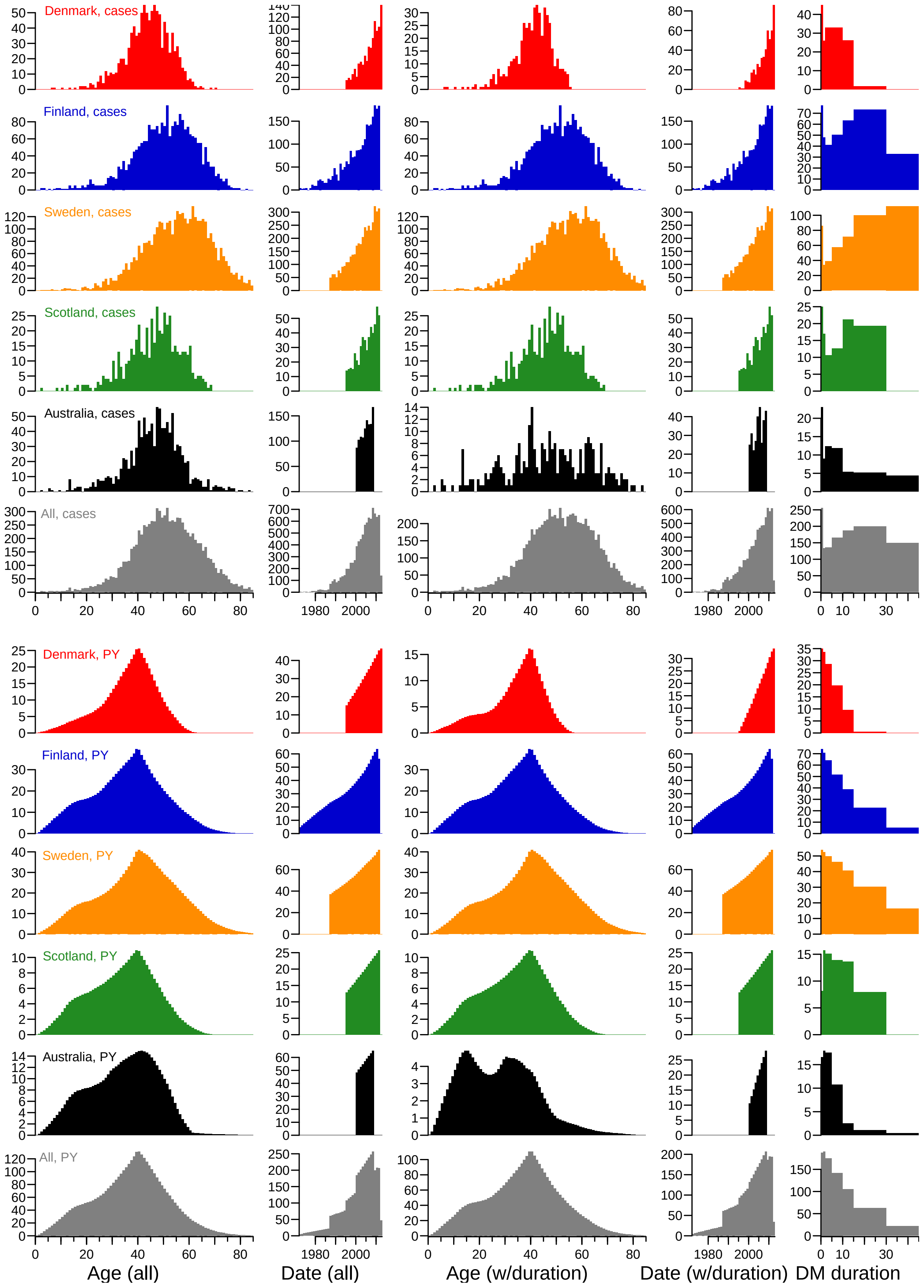

Figure 1: *Distribution of cancer events (top half) and person-years (bottom half) in T1D patients by age and calendar time for all (first two columns), and by age, calendar time and diabetes duration for those with duration of DM known (last three columns).*

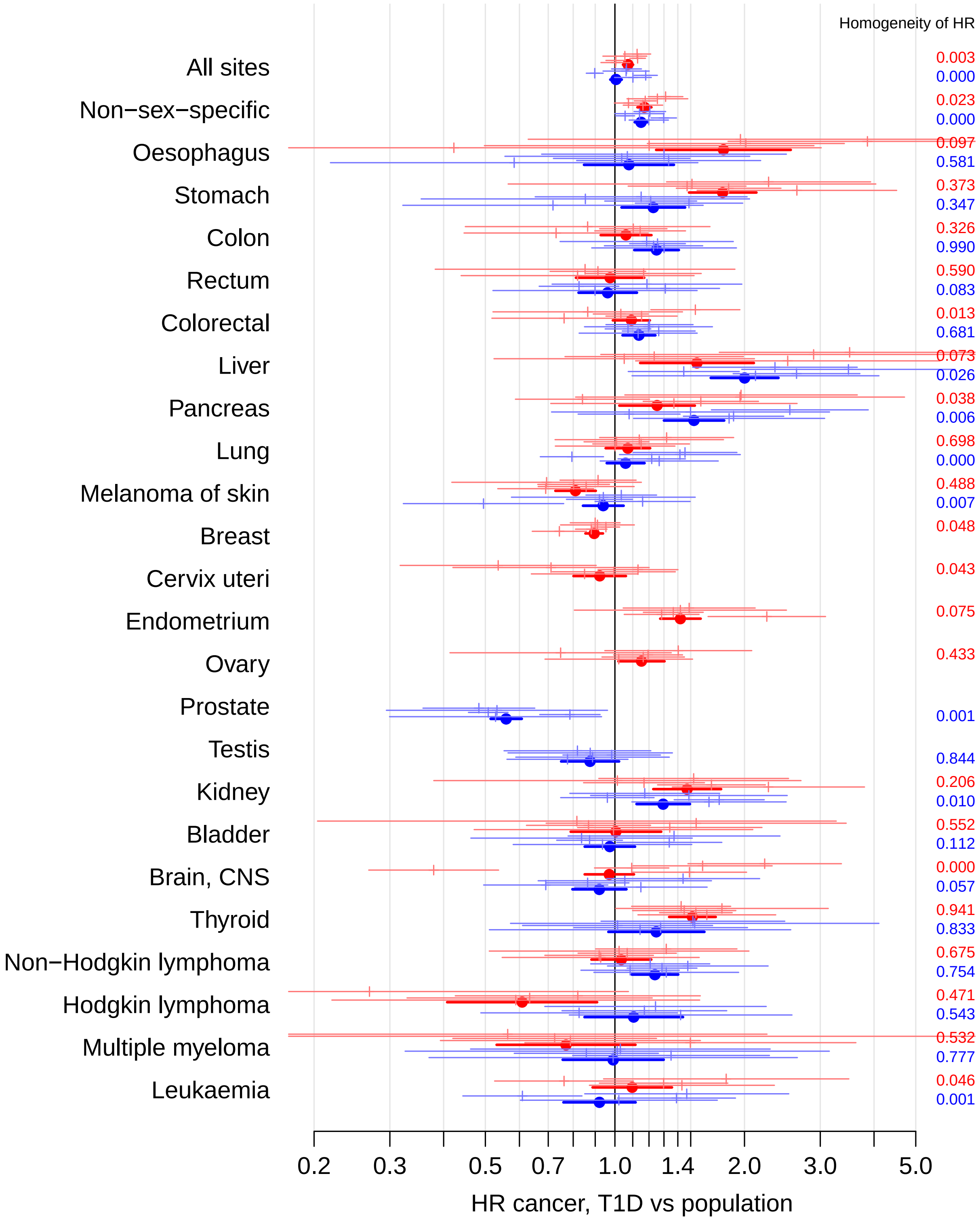

Figure 2: *HR of different cancers from the joint analysis (thick, full color), and the interaction with country (thin, pale color, ordered bottom-up as: DK, FI, SE, SC, AU). Blue: men, red: women.*

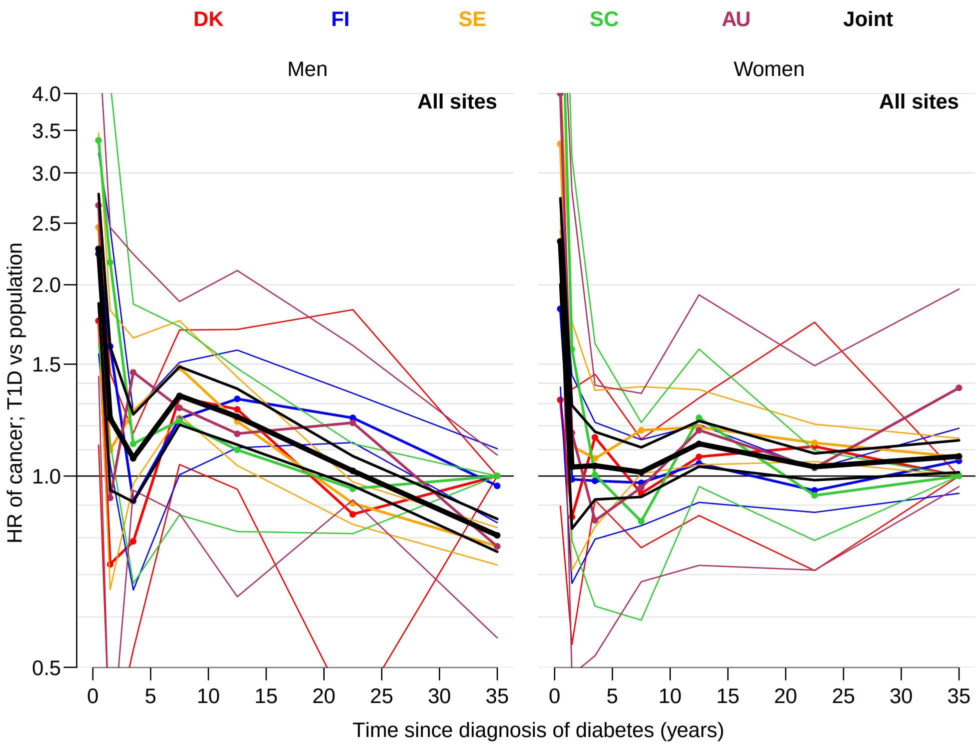

Figure 3: *Hazard ratio of all cancers between T1D patients and the general population, by country*

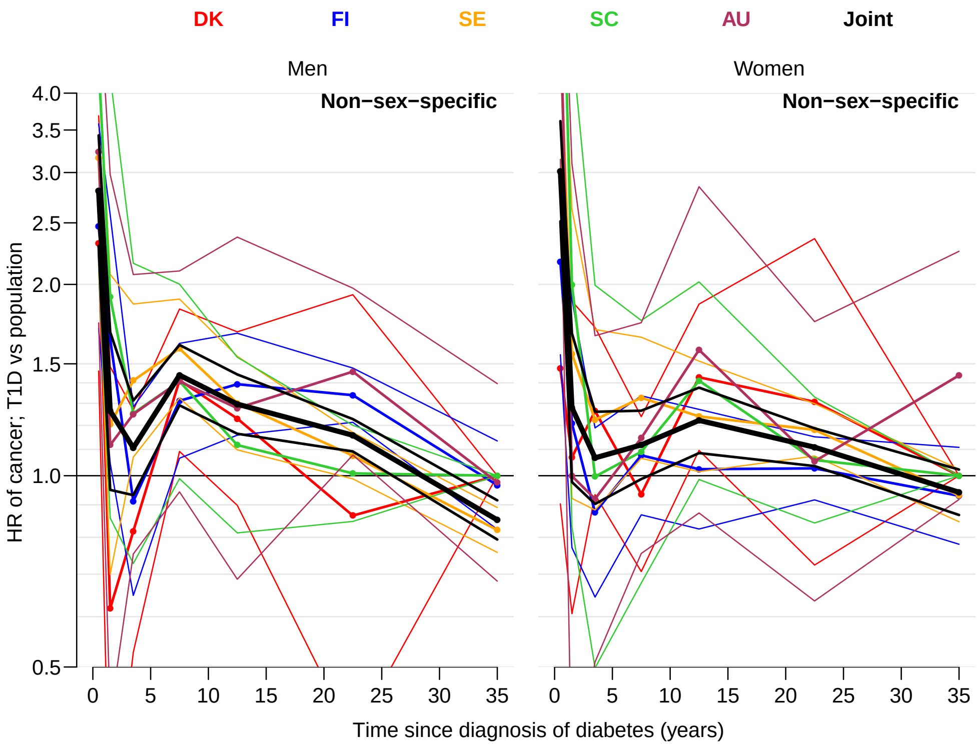

Figure 4: *Hazard ratio of non-sex-specific cancers between T1D patients and the general population, by country*

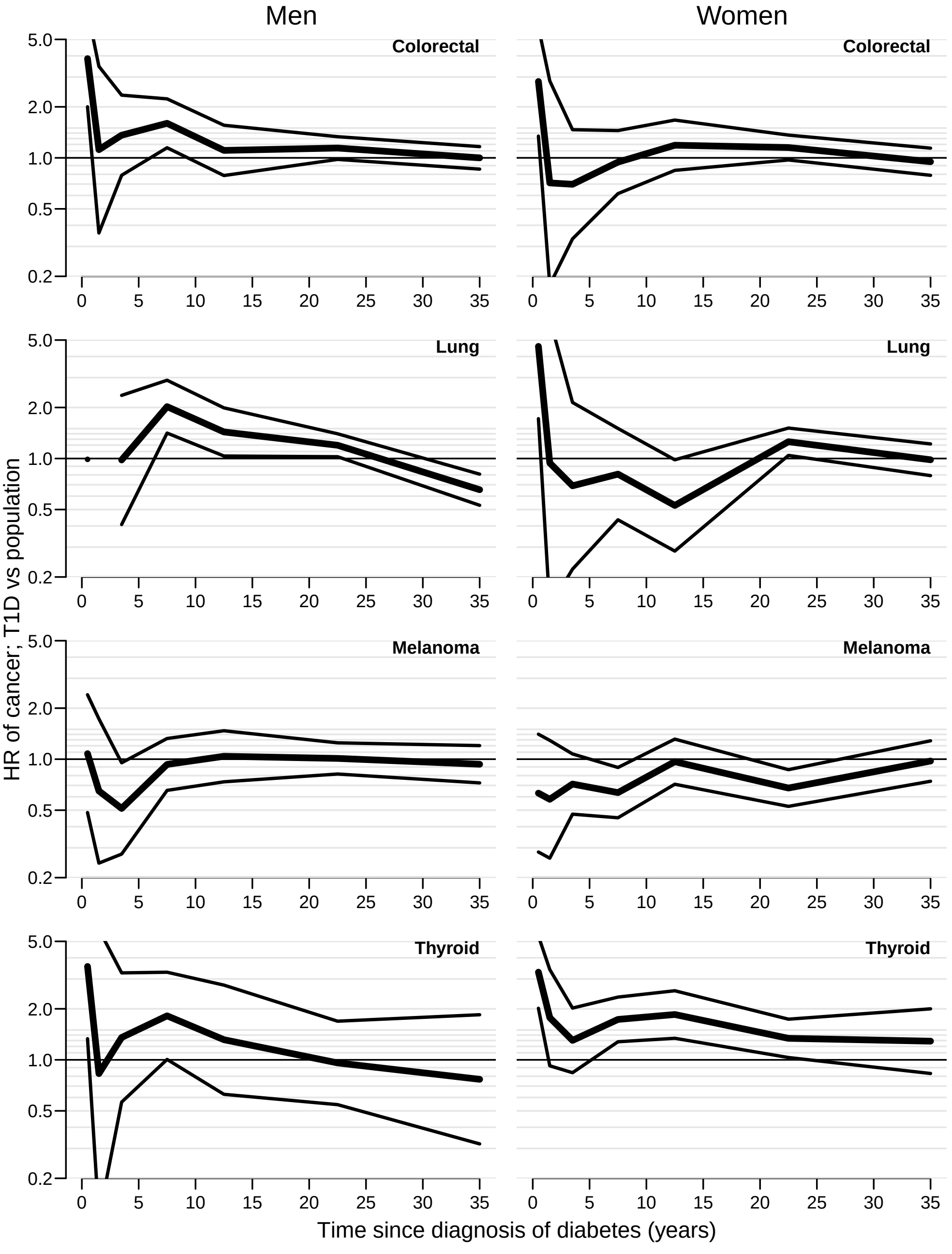

Figure 5: *Hazard ratio of cancer of select sites between T1D patients and the general population. Pooled analysis.*

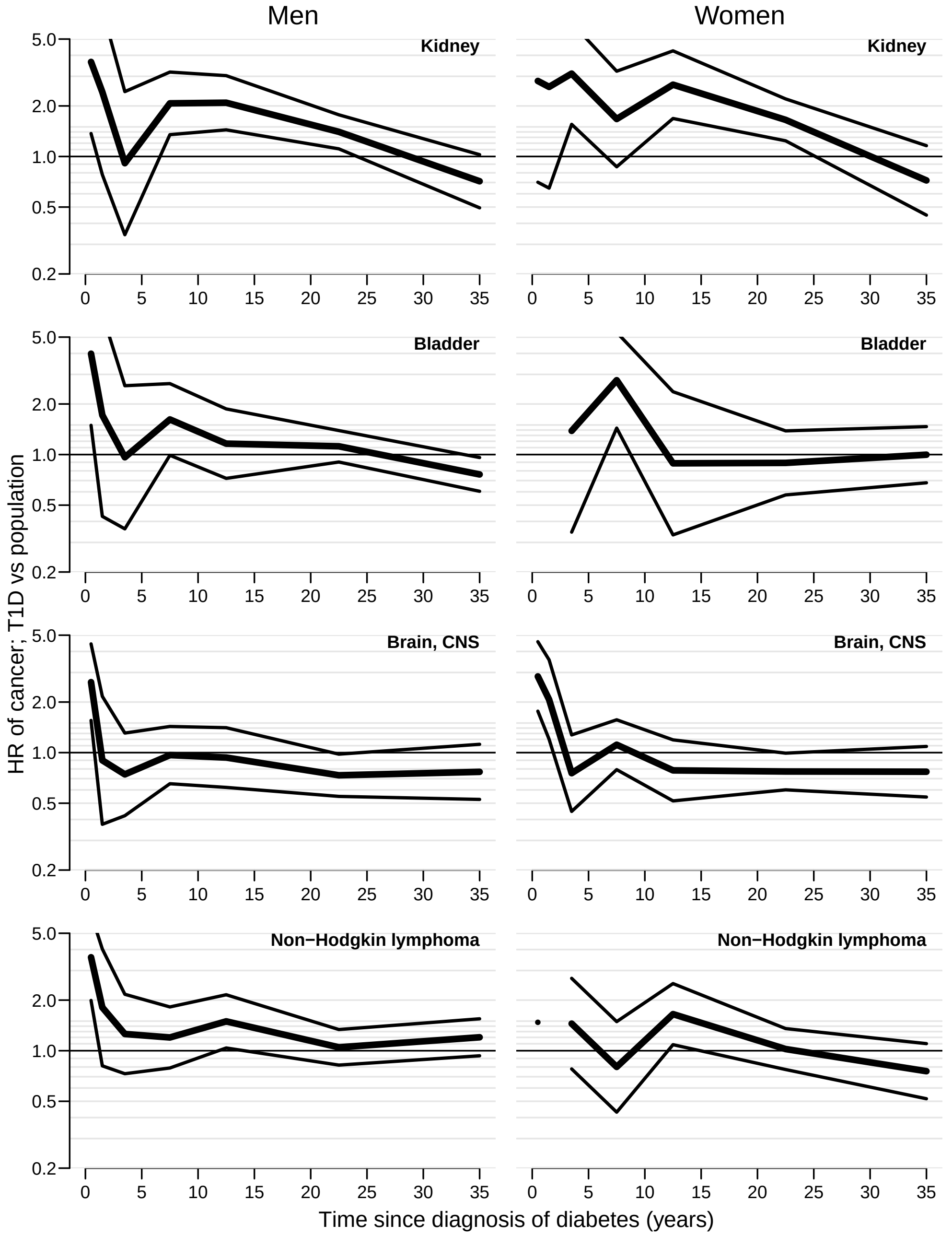

Figure 6: *Hazard ratio of cancer of select sites between T1D patients and the general population. Pooled analysis.*

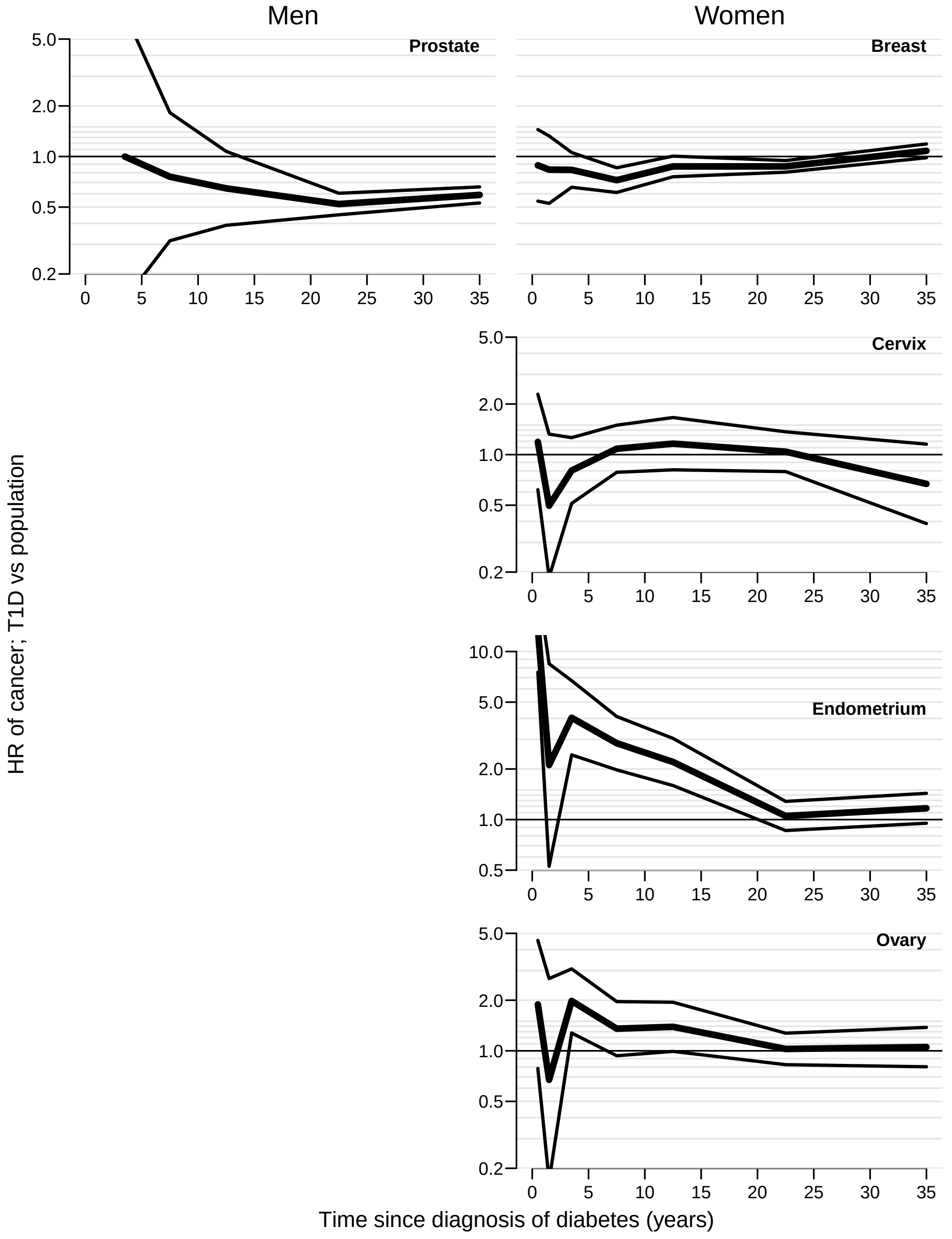

Figure 7: *Hazard ratio of cancer of select sites between T1D patients and the general population. Pooled analysis.*

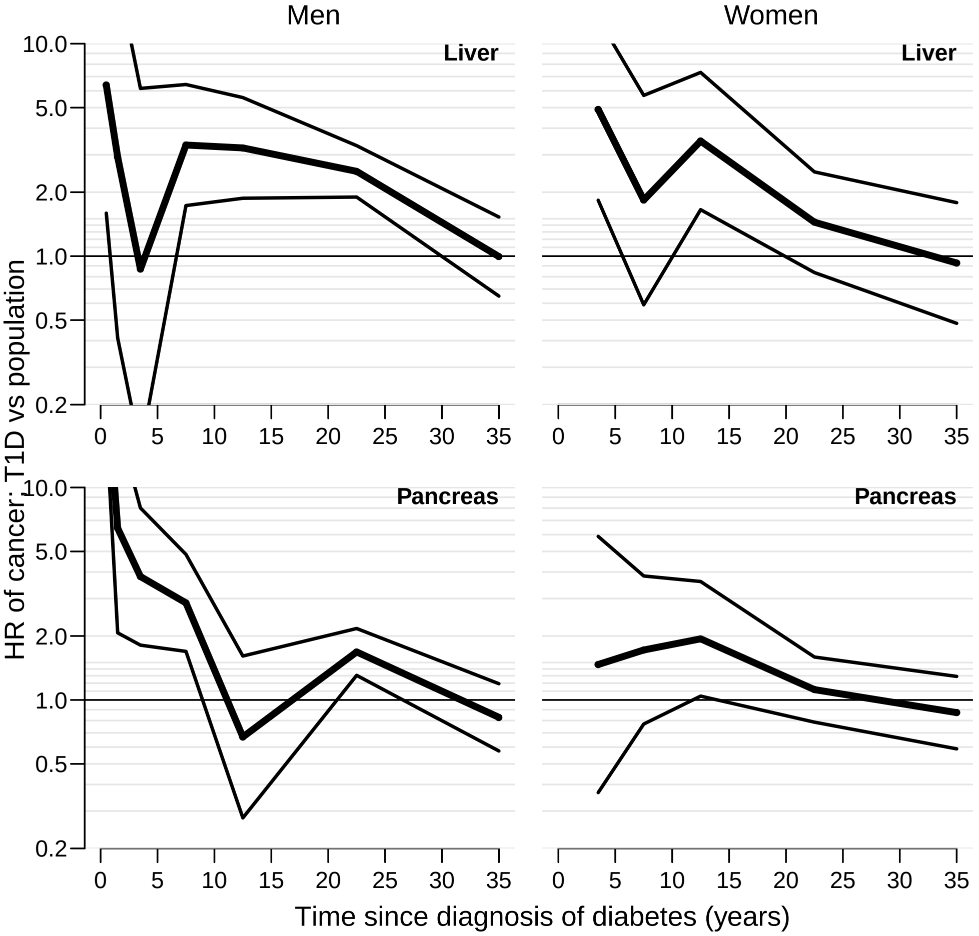

Figure 8: *Hazard ratio of cancer of select sites between T1D patients and the general population. Pooled analysis.*
